# Outcome measures and treatment effectiveness in late onset myasthenia gravis

**DOI:** 10.1186/s42466-020-00091-z

**Published:** 2020-10-30

**Authors:** Francesca Pasqualin, Silvia V. Guidoni, Mario Ermani, Elena Pegoraro, Domenico M. Bonifati

**Affiliations:** 1 Ospedale regionale Ca’ Foncello, Unit of Neurology, 31100 Treviso, Italy; 2 Department of Neuroscience, University of Padova, 35128 Padova, Italy

**Keywords:** MGFA-PIS, MGSTI, Myasthenia gravis, Characterization, Outcome, LOMG

## Abstract

**Background:**

Recently different subtypes of myasthenia gravis (MG) have been described. They differ for clinical features and pathogenesis but the prognosis and response to treatment is less clear. The aim of the study was to evaluate outcome and treatment effectiveness including side effects in late onset MG (LOMG) compared with early onset MG (EOMG).

**Methods:**

We analysed retrospectively 208 MG patients. Clinical features were recorded as well as treatment and side effects. Outcome at the last follow-up was evaluated with MGSTI and MGPIS scales.

**Results:**

The 208 patients included were classified as follow: 36 ocular MG, 40 EOMG, 72 LOMG, 25 thymoma-associated, 14 anti-MuSK and 21 double seronegative. Similar positive outcome was achieved in either early and late onset subgroup. We found pharmacological remission and minimal manifestations at the MGFA-PIS in the 95% and 94,4% of EOMG and LOMG respectively but in LOMG a lower dose of immunosuppressors (MGSTI< 2) was required compared to EOMG (*p* = 0,048). Severe side effects were present in a small percentage of patients in both group but diabetes was more frequent in LOMG vs EOMG (2,2% vs 5%, *p* = 0.017).

**Conclusions:**

Despite LOMG has more comorbidities that might interfere with treatment and outcome, therapeutic management does not seem to differ between EOMG and LOMG. A similar positive outcome was seen in both subgroups but LOMG group seems to require lower doses of medication to control symptoms.

## Introduction

Myasthenia gravis (MG) is an autoimmune disorder due to antibodies against post-synaptic membrane proteins. Different subgroups have been described. The most accepted classification divides myasthenia gravis into the following subgroups: ocular; thymoma-associated; early onset and late-onset generalized with antibodies against acetylcholine receptor (AChR), anti-muscle specific tyrosine kinase (MuSK) positive and double seronegative [1]. They differ for clinical features and probably pathogenesis but prognosis and response to treatment in different subgroups is less clear.

Treatment strategies have changed in time with improved knowledge of pathogenic mechanisms and introduction of new medications. Nowadays treatment includes cholinesterase inhibitors (symptomatic treatment), steroids, immunosuppression and thymectomy.

Therapy is often empirical because very few drugs have undergone randomised double blind clinical trial. Guidelines are mainly based on observational retrospective and prospective studies or expert opinions. Briefly most review and guidelines suggest to start with symptomatic treatment while evaluating thymectomy [2–4]. If minimum manifestation status is not reached, corticosteroid are introduced to the minimal possible dose. Other immunosuppressive drugs (azathioprine first) are considered if significant symptoms are still present or if there is unbearable steroid side effects. As third line treatment in refractory patients mycophenolate mofetil, cyclosporin, tacrolimus, intravenous immunoglobulin (IVIG), plasmaferesis (PLEX), rituximab or more recently eculizumab may be used [5].

However there is not a distinct therapeutic strategy in different subgroups of MG except for anti-MuSK myasthenia where rituximab and plasma-exchange seems to be more effective. It is unknown if early and late onset MG should be treated differently. This may represent a real drawback considering the different pathogenesis and comorbidities. Moreover elderly patients take many drugs that can interfere with MG treatment or be partially contraindicated such as beta-blockers.

To evaluate myasthenia gravis outcome with and without treatment, several scores have been proposed. The Myasthenia gravis foundation of America (MGFA) developed the post-intervention status (MGFA-PIS) to be used for routine follow up and clinical trials. It classifies patient status as remission (complete stable or pharmacological), minimal manifestations or symptomatic and it can also record a change in clinical status [6].

Recently in a double blind prospective study with Rituximab a new score named *Myasthenia gravis status and treatment intensity* (MGSTI) was developed. MGSTI is a composed outcome score mixing the MGFA-PIS and the dose of immunosuppressants the patient is taking [7]. Due to long-term side effects of steroids and other immunosuppressive therapies this score is interesting because it takes in account the burden of treatment and the consequent impact on quality of life. The ultimate goal should be to reach a complete or at least pharmacological remission with a tolerated dose of steroids or immunosuppression.

Only few studies have focused on management and prognosis of late versus early onset MG and have used unbiased scales. They usually outline a good prognosis for LOMG patients with corticosteroid treatment but also highlight the need for more studies to individualize treatment [8–10]. Indeed most trials do not distinguish EOMG versus LOMG, considering only generalized anti- AChR positive myasthenia versus other subgroups.

The aim of our study was to evaluate outcome and treatment effectiveness including side effects with MGFA-PIS and MGSTI in a large group of LOMG in comparison with EOMG.

## Methods

### Subjects

We retrospectively reviewed the clinical records of 208 patients with confirmed MG. The study was approved by local ethical committee and written informed consent was obtained.

Of the entire population of 346 MG patients recorded in our database we excluded patients with missing data (123 patients), other myasthenic syndrome (11 patients) or still unconfirmed diagnosis (4 patients). Two hundred eight patients were then included in the analysis.

Diagnosis of Myasthenia Gravis was confirmed when at least two of the following criteria were present: clinical features typical of MG; serum antibodies against neuromuscular junction proteins; neurophysiological tests (repetitive nerve stimulation or single-fibre electromyography.); Edrophonium/Tensilon test.

We classified patients in the following subgroups: ocular (symptoms strictly ocular for at least 2 years from onset), early onset MG (generalized anti-acetylcholine positive with age at onset < 50 years), late onset MG (generalized anti-acetylcholine positive with age at onset ≥50 years), anti-MuSK MG, double seronegative (anti-AChR and anti-MuSK antibodies absent) and thymoma-associated MG. Demographic and clinical characteristics of each patient were recorded.

Severity at onset and maximum severity were recorded using the MGFA classification.

The following comorbidities were considered: vitiligo, autoimmune hypothyroidism, inflammatory bowel disease (IBD); psoriatic arthritis, systemic lupus erythematosus (SLE), antiphospholipid syndrome, fibromyalgia, gout, parkinson, epilepsy, hypertension, diabetes, cardiovascular disease, atrial fibrillation, asthma, chronic obstructive pulmonary disease (COPD), chronic respiratory failure, psychiatric disease, anxious-depressive syndrome, hepatopathy, diverticulosis, renal failure.

### Outcome scores

We collected the outcome at last follow up visit with two scales: MGFA-PIS and MGSTI. In the MGFA-PIS scale, *remission* is defined as 1 year or longer without signs or symptoms and without any symptomatic (pyridostigmine) treatment. Remission is divided in *complete* (CSR: no pharmacologic treatment at all) or *pharmacologic remission* (PR). *Minimal manifestation* status is defined as minimal signs or symptoms. A patient is considered MM-0 if without any treatment during the last year, MM-1 if some type of immunosuppression is present without symptomatic therapy, MM-2 if the patient has received only low dose of pyridostigmine (< 120 mg/die) for at least 1 year, and MM-3 if the patient has received symptomatic therapy and some form of immunosuppression during the past year.

MGSTI classification identifies 6 subgroups of patients on the basis of clinical remission or minimal manifestations together with the amount of immunotherapy [7]. Level 0 corresponds to complete stable remission without immunotherapy. Level 1 to 3 are MM or PR with an increasing dose of immunotherapy; level 4 and 5 are symptomatic patients (without and with the necessity of IVIG or PLEX respectively) and level 6 are hospitalized patients [7].

### Statistics

Variables, if possible, were expressed as dichotomous variables. The comparison of subgroups of MG was obtained with T-Student Test or non-parametric tests. Differences between dichotomous variables were analyzed with the Chi-quadro test (χ2) or the Fisher exact test.

Variance analysis (ANOVA-Kruskal Wallis test) was used to compare subgroups. Significance was set at *p* < 0,05.

## Results

Two hundred eight patients were included in the study, 104 females and 104 males. The average age was 62,6 ± 15,0 years; higher in males than females (69,3 ± 11,1 vs 55,9 ± 15,4 *p* < 0,0001). The sample included patients with the following subtypes of MG: 36 with ocular MG (14 females, 22 males), 40 EOMG (36 females, 4 males), 72 LOMG (17 females, 55 males), 25 thymoma-associated MG (15 females, 10 males), 14 anti-MuSK positive (10 females, 4 males) and 21 double seronegative (12 females, 9 males).

The mean age at onset was 52,3 ± 19,4 years; higher in males than females (60,8 ± 14,2 vs 43,7 ± 20,2, *p* < 0,00001). As already well described in the literature the age at onset had a bimodal frequency distribution, with a first peak between 30 and 40 years of age and a second peak between 60 and 70 years of age. Females were prevalent in the first peak whereas males in the second one.

Age at onset of EOMG was significantly different when compared with thymoma-associated MG (28,9 ± 11,5 vs 45,0 ± 16,3 *p* = 0,000038), seronegative MG (28,9 ± 11,5 vs 48,2 ± 16,5 *p* < 0,00001) and anti-MuSK positive MG (28,9 ± 11,5 vs 44,8 ± 16,8 *p* = 0,00051).

The mean disease duration was 10,3 ± 9,1 years; higher in females than males (12,2 ± 10,2 vs 8,5 ± 7,5; *p* < 0,00001).

Severity at onset and maximum severity were recorded using the MGFA classification.

34,6% of the sample had an ocular onset. Mild weakness affecting muscles other than ocular was present at onset in 47,5% of patients, of whom 16,3% predominantly affecting limbs (IIA) and 31,2% predominantly bulbar (IIB). 16,4% of the sample had moderate weakness other than ocular at onset of whom 2,9% IIIA and 13,5% IIIB. Just 1% of the sample had a severe involvement at onset (0,5% IVA, 1% IVB). (Table 1).

**Table 1:** Clinical characteristics of the sample

Type of MG (n° pts)		OCULAR (36)	EOMG (40)	LOMG (72)	THYMOMA (25)	SN (21)	ANTI-MUSK (14)	Total (208)
sex Female/male		14 /22	36 /4	17 /55	15 /10	12 /9	10 /4	104 /104
Average age (years)		68,3 ± 10.9	44,2 ± 10,7	73,5 ± 8,5	59,4 ± 14,4	58,3 ± 10,9	57,4 ± 8,9	62,6 ± 15,0
Average age at onset (years)		60,8 ± 15,3	28,9 ± 11,5	66,8,1 ± 9,3	45,0 ± 16,3	48,2 ± 16,5	44,8 ± 16,8	52,3 ± 19,4
Disease duration		7,4 ± 7,7	16,0 ± 11,3	6,7 ± 4,0	14,4 ± 9,4	10,1 ± 9,2	12,6 ± 12,5	10,3 ± 9,1
Severity at Onset (MGFA)	I	36	1 (2,5%)	23 (31,9%)	2	10	0	72 (34,6%)
	IIA/IIB	0	11 (27,5%)/14 (35%)	11 (15,3%)/26 (36,1%)	5/12	5/12	2 /9	34 (16,3%)/65 (31,2%)
	IIIA/IIIB	0	1 (2,5%)/11 (27,5%)	2 (2,8%)/10 (13,9%)	1/5	1/5	1/1	6 (2,9%)/25 (12,1%)
	IVA/IVB	0	1 (2,5%)/1 (2,5%)	0	0	0	0/1	1 (0,5%)/2 (1%)
Worst severity during the course of the disease (MGFA)	I	36	0	0	0	0	0	36 (17,3%)
	IIA/IIB	0	5 (12,5%)/11 (27,5%)	8 (11,1%)/25 (34,7%)	4/4	7/7	1/4	25 (12,1%)/51 (24,5)
	IIIA/IIIB	0	2 (5%)/13 (32,5%)	3 (4,2%)/24 (33,3%)	2/11	1/5	0/4	8 (3,8%)/57 (27,4%)
	IVA/IVB	0	2 (5%)/3 (7,5%)	2 (2,8%)/7 (9,7%)	0/3	0/1	0/3	4 (1,9%)/17 (8,2%)
	V	0	4 (10%)	3 (4,2%)	1	0	2	10 (4,8%)
thymic hyperplasia (histology/thymectomized)		2/3	34/34	4/4	0/25	4/5	0/1	44/ 72
N. of comorbidity	0	9 (25%)	20 (50%)	7 (9,7%)	8 (32%)	3 (14,3%)	3 (21,4%)	50
	1–2	24 (66,7%)	14 (35%)	39 (54,2%)	10 (40%)	17 (80,9%)	9 (64,3%)	113
	3–4	3 (8,3%)	6 (15%)	22 (30,6%)	6 (24%)	1 (4,8%)	2 (14,3%)	40
	5–6			4 (5,6%)	1 (4%)			5

Abbreviations: *F* Females, *M* Males, *T* Total, *EOMG* Early onset myasthenia gravis, *LOMG* Late onset myasthenia gravis, *MUSK* Muscle specific tyrosine kinase, *SN* Double seronegative

An ocular onset was more frequent in LOMG than EOMG (31,9% vs 2,5% *p* = 0,00076).

Worst severity during the course of disease was registered with the MGFA classification. (Table 1).

Worst severity was not significantly different between LOMG and EOMG.

### Therapy at the last follow-up visit

At the last follow up visit, 2,5% of EOMG patients and 4,2% of LOMG patients were in complete non-pharmacological remission while 2,5% and 11,1% respectively were on symptomatic treatment. Corticosteroid monotherapy was the treatment in 52,5% of EOMG patients and in 48,6% of LOMG patients. Other immunosuppressive monotherapy was used in 12,2% and 9,9% of early and late onset MG respectively. 25% of EOMG patients and 22.2% of LOMG patients were taking steroids plus an immunosuppressive drug. Chronic IVIG with an immunosuppressive drug with or without corticosteroids were used in 4,2% of LOMG patients. Chronic PLEX with corticosteroids and an immunosuppressive drug were used in 2,4% of EOMG patients.

As a whole 47,1% of patients used corticosteroids for less than 5 years mainly in LOMG group compared with EOMG (56,9% vs 27,5% respectively, *p* = 0,003); 23,6% of the sample used corticosteroids for a period between 6 and 9 years and 22,6% for more than 10 years mainly in EOMG compared with LOMG (47,5% vs 11,1% respectively *p* < 0,0001). Corticosteroids were never used in 9.1% of patients, mainly in the LOMG subgroup (8,3% vs 2,5%, p 0,22). The overall number of patients who took corticosteroids for less than < 5 years or never was significantly higher in the LOMG subgroup (*p* = 0,00034).

In the EOMG group azathioprine was used for less than 5 years in 37% of patients and longer in 20% while 40% of patients never used azathioprine. In the LOMG group azathioprine was used for less than 5 years in 26,8% of patients and longer in 18,3% while 56,3% of patients never used azathioprine.

### Treatment outcome measures at the last follow-up

At the last follow-up visit, 90 patients (43,3%) were on pharmacological remission. Fifty four patients (26%) had a MGFA-PIS (post intervention status) of MM-3, 24 patients (11,5%) of MM-1, 18 patients (8,7%) of MM-2, 3 patients (1,4%) a MM-0, 7 patients (3,4%) were on complete stable remission (CSR), and 12 patients (5,8%) had refractory symptoms (Table 2).

**Table 2:** MGFA PIS and MGSTI at last follow up

**MGFA-PIS**								
**Type of MG**	CSR/ MM-0	MM-1	MM-2	MM-3	PR	Symptomatic		Total
**Ocular**	4 (11,1%)	6 (16,7%)	9 (25%)	3 (8,3%)	13 (36,1%)	1 (2,8%)		36
**EOMG**	1 (2,5%)	5 (12,5%)	1 (2,5%)	15 (37,5%)	16 (42,5%)	2 (5%)		40
**LOMG**	3 (4,2%)	5 (6,9%)	5 (6,9%)	23 (31,9%)	32 (45,8%)	4 (5,6%)		72
**Thymoma**	1 (4,0%)	1 (4,0%)	–	7 (28%)	13 (52%)	3 (12%)		25
**Sieronegative**	1 (4,8%)	5 (23,8%)	3 (14,3%)	5 (23,8%)	7 (33,3%)	-		21
**Anti-MUSK**	-	2 (14,3%)	-	1 (7,1%)	9 (64,2%)	2 (9,5%)		14
**Total**	10 (4,8%)	24 (11,5%)	18 (8,7%)	54 (26%)	90 (44,2%)	12 (5,8%)		208
**MGSTI**								
**Type of MG**	0	1	2	3	4	5	6	Total
**Ocular**	16 (44,4%)	9 (25%)	9 (25%)	1 (2,8%)	1 (2,8%)			36
**EOMG**	2 (5,0%)	7 (17,5%)	16 (40%)	13 (32,5%)		2 (5,0%)		40
**LOMG**	11 (15,3%)	20 (27,8%)	26 (36,1%)	11 (15,3%)	1 (1,4%)	2 (2,8%)	1 (1,4%)	72
**Thymoma**	1 (4,0%)	7 (28,0%)	13 (52,0%)	1 (4,0%)		2 (8,0%)	1 (4,0%)	25
**Sieronegative**	7 (30,4%)	3 (13,0%)	6 (26,1%)	4 (17,4%)				23
**Anti-MUSK**		2 (14,3%)	8 (57,1%)	2 (14,3%)		2 (14,3%)		14
**Total**	37 (17,9%)	48 (23,2%)	78 (37,5%)	32 (15,5)	2 (1,0%)	8 (3,9%)	2 (1,0%)	208

Abbreviations: *CSR* Complete stable remission, *EOMG* Early onset myasthenia gravis, *LOMG* Late onset myasthenia gravis, *MM* Minimal manifestations, *MUSK* Muscle specific tyrosine kinase, *PR* Pharmacologic remission

No statistically significant association was found between the subtype of MG and MGFA-PIS scale.

The association of MGSTI scale and subtype of MG is summarized in Table 2. Using Kruskal-Wallis ANOVA by ranks a statistically significant association was found (*p* < 0.0001). Overall outcome did not differ between LOMG and EOMG but LOMG patients reached MM or PR status with lower dose of immunosuppressors (MGSTI < 3) (*p* = 0,048).

A mild inverse correlation was found between MGSTI and age (Spearman R = − 0,26, *p* = 0,0002), and between MGFA-PIS and age (Spearman R = -0,16, *p* = 0,02) while a mild direct correlation was found between MGFA-PIS and MGSTI (Spearman R = 0,35, *p* = 0,02).

A chi-square test of independence was performed to examine the relation between outcome at last follow up and severity at onset. The relation between these variables was not significant (*p* = 0,49).

A significant dependence was found between MGSTI at last follow up and worst severity in the course of the disease (*p* = 0,0006).

When we evaluate the severity of the course of the disease (crisis or exacerbation) measured by the number of acute IVIG or PLEX cycles no significant difference between EOMG and LOMG was found as well as with other subgroups but ocular and seronegative MG (*p* < 0,0001).

Severe side effects were rare. A direct correlation between number of side effects over the years and duration of the disease was found (R = 0,314 *p* < 0,00001). A linear correlation came out between number of side effects and duration of corticosteroid therapy (R = 0,33, *p* < 0,00001) but not with duration of azathioprine treatment.

**Fig. 1:**
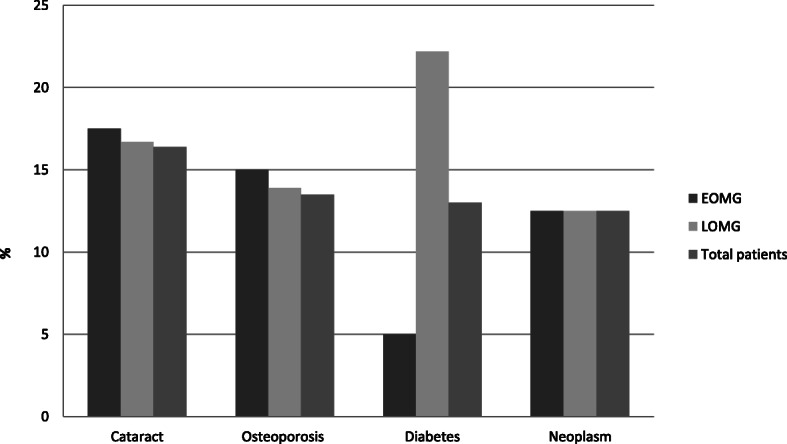
Side effect. Abbreviations: EOMG = early onset myasthenia gravis, LOMG = late onset myasthenia gravis

The most frequent side effects were cataract, osteoporosis, diabetes and neoplasms. Except for diabetes, which was more frequent in LOMG patients, prevalence of cataract, osteoporosis and neoplasms did not differ between EOMG and LOMG patients *(*Fig. 1*).*

LOMG patients had a higher number of comorbidities compared to EOMG (2,18 ± 1,34 vs 0,95 ± 1,22 *p* < 0,00001). Autoimmune diseases were more frequent among EOMG patients (35,0% vs 13,9%, *p* = 0,04) with autoimmune hypothyroidism the most common. Hypertension, atrial fibrillation, cardiovascular disease and diabetes were more frequent in LOMG subgroup. Anxious and depressive disorder were equally represented in both LOMG and EOMG. A summary of comorbidities in the MG subgroups is listed in Supplemental materials*.*

## Discussion

Treatment strategies in MG changed over time with the introduction of new drugs and deeper knowledge of pathogenesis but their use is often empirical and not based on clinical trials. Only recently we have also understood that there are many “myasthenias” but it is not known if different subgroups should be treated differently.

Literature on this issue is poor, especially about treatment of early versus late onset generalized MG. Moreover, the majority of clinical trials or observational studies focus on generalized or ocular acetylcholine receptor antibodies positive MG, but rarely on EOMG and LOMG.

For long time LOMG has been considered to have a worst prognosis compared to EOMG. A retrospective analysis of Besta Institute in Milan showed that a higher chance of CSR was associated with the following factors: age at onset < 40 years, clinical stage at maximal worsening and thymic histology [11]. However more recent papers underline an increased chance for good outcome in LOMG that has been attributed to a more aggressive treatment [9]. In a multivariate analysis of 268 MG patients regardless of antibody status, Andersen et al. showed that an age at onset after 50 years increased the probability of optimal outcome. The authors highlighted that 87% of patients over 50 years of age were treated with at least an immunosuppressant. In another Norwegian nationwide population analysis of total drug treatment in MG, only 56% of patients over 50 years of age were treated with immunoactive drugs. However the study considered the age at recruitment and not the age at onset thereby early and late onset MG patients were combined in the group above 50 years [12].

In our study therapeutic management did not differ between EOMG and LOMG. Both groups had a positive outcome in around 95% of patients but the prevalence of patients who used corticosteroid for less than 5 years or never was higher in LOMG in comparison with EOMG subgroup. Slightly less than 10% of LOMG patients have never used corticosteroids. This can be due to the benign course of the disease but also to the presence of comorbidities.

Outcome in such a disease as myasthenia should consider symptoms but also the burden of therapy. MGSTI is a new scale that has never been used to compare outcome in MG subtypes but has the advantage to include the amount of drug types and dosages other than clinical status. When we compare outcome in EOMG and LOMG using MGSTI at the last follow up, it came out clearly that a good outcome was achieved in LOMG with lower doses of medication.

It is possible that considering the different pathogenesis, LOMG has a lower level of activity [13, 14]. In EOMG the loss of tolerance seems to derive from an intrathymic process, also defined as thymitis, that drives an hyperactivation of B cells with abnormal production of anti-AChR antibodies [15]. In LOMG instead, thymus atrophy together with the presence of striational self-antibodies suggest a more widespread autoimmunity due to immunosenescence [15, 16] but the concentration of AChR antibodies is lower [13]. On the other hand in our study the severity of the course of the disease seems similar between EOMG and LOMG when we considered the number of acute IVIG or PLEX cycles.

A correlation between duration of the disease and amount of side effects came out. In our study the percentage of patients who took corticosteroids for more than 10 years was four fold in EOMG in comparison with LOMG. Cataract, osteoporosis, diabetes and neoplasia were the most frequent.

Osteoporosis was recorded as a side effect based on densitometric diagnosis. However most patients took vitamin D or bisphosphonates since osteopenia was detected. Moreover the prevalence of osteoporosis should be compared with an age-matched population. In general population the onset of primary osteoporosis is typically between the sixth and seventh decade. Thereby in elderly, prevalence of osteoporosis combines primary and secondary osteoporosis, while in young patients is mostly secondary to prolonged corticosteroid treatment. In the same way cataract had an equal prevalence in the two groups, however in general population it would be unusual to find the same prevalence in young people.

DM was more frequent in LOMG patients but this is probably due to an higher incidence with age.

An higher incidence of neoplasms in myasthenic patients compared to the general population has never been demonstrated [17]. A warning, considering its mechanism of action, is present for azathioprine especially for non melanomas cutaneous tumors [18]. We didn’t found any organ specific prevalence of neoplasms. Moreover between the 6 patients that developed a cutaneous tumor, only 50% were on azathioprine, and just one patient for more than 5 years. This seems to suggest no relation between azathioprine and development of neoplasms but further larger prospective studies should be conducted on the long term effect of different immunosuppressive therapies.

The goal of care in MG is to achieve maximal clinical benefit on low-dose immunosuppressants [3]. In our study response to treatment and outcome were evaluated with the MGSTI score. Although it was developed for Rituximab trial and it has not been used in other cohort studies, it reflects the MG treatment aim [7]. We don’t know if it can be useful in everyday clinical practice and, as the authors suggest, it should be validated in other MG cohorts and prospective MG trials.

In our study it showed to be reliable and with higher sensibility than MGFA-PIS in picking up differences between subgroups. It seems particularly important to consider the burden of immunotherapy when subgroups with similar clinical features but different age are compared.

The retrospective nature of the study did not allow to test MGSTI scale over time or to evaluate the time needed to reach a positive outcome in different MG subtypes.

## Conclusions

Despite LOMG patients have more comorbidities that might interfere with treatment, myasthenia management did not differ between EOMG and LOMG, with similar positive outcomes in most patients. LOMG subgroup required lower doses of corticosteroids and immunosuppressive drugs to keep the disease under control. The prevalence of side effects did not differ between EOMG and LOMG patients but in EOMG side effects such as cataract, diabetes and osteoporosis were more frequent compared to age-matched population, probably due to the longer duration of corticosteroid therapy.

## Supplementary information

**Additional file 1.**

## Data Availability

The data that support the findings of this study are available from the corresponding author upon reasonable request.

## Abbreviations

AChR: Acetylcholine receptor
AZA: Azathioprine
COPD: Chronic obstructive pulmonary disease
CSR: Complete stable remission
EOMG: Early onset myasthenia gravis
IBD: Inflammatory bowel disease
IVIG: Intravenous immunoglobulin
LOMG: Late onset myasthenia gravis
MG: Myasthenia gravis
MGFA-PIS: Myasthenia gravis foundation of America- Post intervention status
MGSTI: Myasthenia gravis status and treatment intensity
MM: Minimal manifestation
MMF: Mycophenolate mofetil
MTX: Methotrexate
MuSK: Muscle-specific tyrosine kinase
PLEX: Plasma-exchage
PR: Pharmacologic remission
SLE: Systemic lupus erythematosus
