# Supplementary material for: Outcome measures and treatment effectiveness in late onset myasthenia gravis
Source: Neurol Res Pract. 2020 Oct 30;2:45. doi: 10.1186/s42466-020-00091-z (PMC7650071; doi:10.1186/s42466-020-00091-z)
Supplement: Supplementary file 1 — Additional file 1. [file 42466_2020_91_MOESM1_ESM.docx]

A. Comorbidities

|  | OCULAR​ | EOMG​ | LOMG​ | THYMOMA​ | SN | MUSK+​ | Total ​ |
| --- | --- | --- | --- | --- | --- | --- | --- |
| Autoimmune dis. § | 6 (16,7%) | 13 (32,5%) | 10 (13,9%) | 7 | 3 | 2 | 41(19,7%) |
| Fibromyalgia | 0 | 1 (2,5%) | 3 (4,2%) | 1 | 1 | 0 | 6 (2,9%) |
| Gout | 0 | 0 | 1 (1,4%) | 0 | 0 | 0 | 1 (0,5%) |
| Parkinson | 0 | 0 | 3 (4,2%) | 0 | 0 | 1 | 4 (1,9%) |
| Epilepsy | 0 | 2 (5%) | 2 (2,8%) | 1 | 0 | 0 | 5 (2,4%) |
| Hypertension | 15 (41,7%) | 5 (12,5%) | 42 (58,3%) | 10 | 4 | 2 | 78 (37,5%) |
| Diabetes | 7 (19,4%) | 1 (2,5%) | 19 (26,4%) | 1 | 0 | 0 | 28 (13,5%) |
| Cardiovascular dis. | 3 (8,3%) | 1 (2,5%) | 19  (26,4%) | 4 | 0 | 1 | 28 (13,5%) |
| AF | 2 (5,6%) | 1 (2,5%) | 15 (20,8%) | 3 | 1 | 0 | 22 (10,6%) |
| Asthma | 2 (5,6%) | 1 (2,5%) | 3 (4,2%) | 1 | 2 | 0 | 9 (4,3%) |
| COPD | 2 (5,6%) | 0 | 4 (5,6%) | 1 | 2 | 0 | 9 (4,3) |
| Chronic respiratory failure | 2 (5,6%) | 0 | 3 (4,2%) | 3 | 0 | 4 | 12 (5,8%) |
| Psychiatric dis. | 0 | 2 (5%) | 4 (5,6%) | 1 | 0 | 1 | 8 (3,9%) |
| Anxious depressive syndrome | 5 (13,9%) | 9 (22,5%) | 18 (25,0%) | 4 | 6 | 5 | 47 (22,6%) |
| Hepatopathy | 0 | 1 (2,5%) | 1 (1,4%) | 1 | 0 | 1 | 4 (1,9%) |
| Diverticulosis | 0 | 1 (2,5%) | 5 (6,9%) | 2 | 1 | 0 | 9 (4,3%) |
| Renal failure | 0 | 0 | 5 (6,9%) | 0 | 0 | 0 | 5 (2,4%) |

**§**Autoimmune disorders were as follows: vitiligo (1 EOMG and 1 thymoma), hypothyroidism (5 ocular, 7 EOMG, 7 LOMG, 4 thymoma, 2 SN, 2 anti-MusK), Inflammatory bowel disease (1 ocular, 2 EOMG, 2 LOMG), Psoriatic arthritis (1 EOMG, 1 LOMG), antiphospholipid syndrome (1 EOMG)

Abbreviations: AF= atrial fibrillation, COPD= chronic obstructive pulmonary disease, dis.= disease , EOMG= early onset myasthenia gravis, LOMG= late onset myasthenia gravis, MUSK+ = anti muscle-specific tyrosine kinase antibodies positive, SN= seronegative
